# A Little Neutrophil Predominance May Not Be a Harbinger of Death: Clinical and Laboratory Characteristics of Meningitis in Jordan

**DOI:** 10.7759/cureus.29864

**Published:** 2022-10-03

**Authors:** Husam A Abuhayyeh, Belal M Al Droubi, Jowan M Al-Nusair, Bashar M Malkawi, Lana K Haddad, Nour M Abed Alfattah, Jamaledin H Abu Ghaida

**Affiliations:** 1 Department of Pediatrics and Neonatology, Faculty of Medicine, Jordan University of Science and Technology, Irbid, JOR; 2 Department of Anatomy, Faculty of Medicine, Jordan University of Science and Technology, Irbid, JOR

**Keywords:** pretreatment, antibiotics, epidemiology, jordan, aseptic, enterovirus, cns infection, meningitis

## Abstract

Background

This study aims to evaluate the clinical features, laboratory findings, and outcomes of children and adults diagnosed with meningitis in Jordan.

Methodology

This is a retrospective chart review study that targeted patients diagnosed with meningitis at King Abdullah University Hospital, a tertiary care center in Northern Jordan, from March 21, 2015, to March 31, 2019. Patients were included in this study if they were older than 28 days and had no risk factors for meningitis.

Results

A total of 169 patients met the inclusion criteria. Males were overrepresented (67%) and were significantly younger than females (6 vs. 17 years, p = 0.01). Positive meningeal signs were not predictive of greater cerebrospinal fluid leukocytosis (p = 0.348), and they did not provide sufficient sensitivity to be used as screening tools. The most common etiology was aseptic (49%), followed by enterovirus (43%), while bacterial meningitis was an uncommon diagnosis (3.5%). Nearly half of the patients took antibiotics prior to their hospital presentation. During in-hospital admission, six patients died, four of whom had bacterial and two had aseptic meningitis. Enteroviral meningitis showed neutrophil predominance in 44% of cases on lumbar puncture and had a higher neutrophil proportion compared to aseptic meningitis (p = 0.026). *Streptococcus pneumoniae* was the most common bacterial etiology identified.

Conclusions

Meningitis in Jordan is most commonly of aseptic and enteroviral origin, and these etiologies carry significantly more favorable outcomes compared to bacterial meningitis. Enteroviral meningitis displays a higher percentage of neutrophils in cerebrospinal fluid compared to aseptic meningitis. *S. pneumoniae* is the leading cause of bacterial meningitis. Slight neutrophil predominance above half is a weak predictor of bacterial meningitis due to the small contribution of bacteria as a cause among enteroviruses and aseptic etiologies.

## Introduction

Meningitis is defined as an inflammatory disease that involves the leptomeninges and cerebrospinal fluid (CSF) within the subarachnoid space and can affect people of different ages [1]. Most commonly, the underlying etiology is infectious. Although less often, meningitis may be caused by a non-infectious etiology, such as systemic diseases, drug-related adverse events, and neoplasia [2].

Meningitis is most commonly caused by viruses, with the most popular being enterovirus [3]. As for bacterial etiologies, the literature has demonstrated significant changes during the past years in the overall incidence and etiologies of bacterial meningitis, which was mainly driven by the remarkable vaccination efforts worldwide [4,5]. Due to the grand success of widespread vaccination against *Haemophilus influenzae* type b (Hib), there has been a decrease in its incidence, which has cleared the path for other, less commonly vaccinated-against bacteria, such as *Streptococcus pneumoniae*, *Neisseria meningitidis*, and others to take the lead [4,5].

Advancements in biomedical technology and the introduction of prophylactic conjugated vaccines against these leading etiologies have resulted in a significant favorable shift in the epidemiologic profile where these vaccines were implemented [5,6]. Unfortunately, however, lower- and middle-income countries with lagging vaccination programs are dealing with the burden of high bacterial meningitis incidence and mortality rates [6,7].

Research on the clinical presentation and various etiologies of meningitis in Jordan is scarce. Moreover, knowledge of these aspects can aid clinicians in the diagnosis and management of this disease, in addition to guiding future public health decisions through a better understanding of the local epidemiology in Jordan. In this study, we aim to describe the epidemiology of this disease in Jordan, highlight the clinical features and assess the reliability of various signs and symptoms of meningitis, investigate the relative contribution of meningitis-causing microorganisms, and compare their CSF findings.

## Materials and methods

Study design

This retrospective study involved data collection from King Abdullah University Hospital (KAUH), a tertiary care hospital in northern Jordan. The data was extracted from KAUH’s electronic healthcare records system. This included all visits to the study center labeled as meningitis from March 21, 2015, to March 31, 2019.

Ethical approval

This study was approved by the Institutional Review Board ethical committee (IRB #51/117/2018) at Jordan University of Science and Technology.

Patients and inclusion criteria

We included patients older than 28 days with a clinical picture suggestive of meningitis (headache, fever, vomiting, seizures, confusion or drowsiness, meningeal signs, photophobia, or phonophobia). We included patients without any central nervous system (CNS)-related anatomical risk factors or immunodeficiency status. The included patients underwent a lumbar puncture that showed a CSF leukocyte count ≥8 cells/μL.

**Figure 1:**
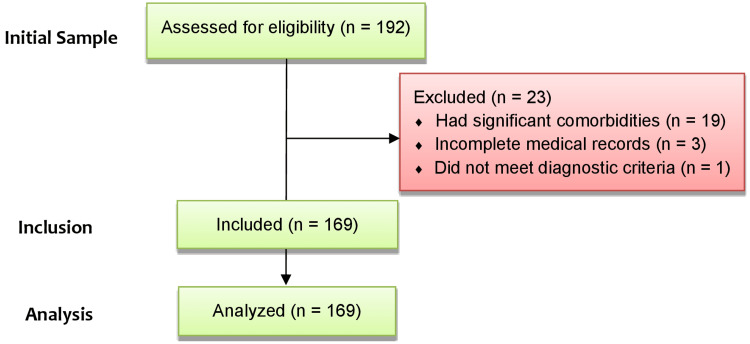
The inclusion and exclusion process of the studied sample.

During the initial screening process, 192 patients were evaluated. Subsequently, using our criteria, a total of 23 patients were excluded. Details are shown in Figure 1. The patients excluded for having CNS-related anatomical risk factors and immunological comorbidities (n = 19) had the following conditions: recent head trauma, fracture or head and neck surgery within the past year (n = 7), presence of ventriculoperitoneal shunt (n = 1), immunodeficiency (n = 8), and craniospinal malformations (n = 3).

Defining the variables of interest

Bacterial meningitis was defined by the presence of suggestive symptoms and a positive CSF or blood culture of a known meningitis-causing organism. Viral meningitis was defined by the same and a positive CSF polymerase chain reaction (PCR). Aseptic meningitis was used to label cases of unknown etiology, where neither bacterial nor viral etiologies were identified using the aforementioned methods. This was a heterogeneous group as it potentially included untested viruses, systemic diseases with meningeal involvement, neoplastic meningitis, and drug-induced meningitis.

The temperature readings of the patients were taken at presentation in the Emergency Room (ER) and were considered elevated at >37.5°C. History of fever was recorded as positive if the patients or their caretakers reported fever or chills, regardless of whether documented by a thermometer or not. Mortality was defined by all-cause, in-hospital deaths which occurred during the hospital stay.

Investigations

All included patients had CSF samples taken and analyzed. This included CSF protein, glucose, leukocytes, erythrocytes, CSF culture, and gram staining. CSF PCR was done for most patients; enterovirus (n = 158), herpes simplex virus (HSV) I (n = 155), HSV II (n = 155), Epstein-Barr virus (EBV) (n = 61), cytomegalovirus (CMV) (n = 60), and tuberculosis (n = 57).

Statistical analysis

Continuous variables were expressed either as mean ± standard deviation (SD) or as percentages. Where more appropriate due to the skewness of the data, the median and interquartile range (first to third quartile) were reported. Where inferential statistical analyses were performed between continuous variables, the Mann-Whitney U test was used, which is a non-parametric alternative to the independent-samples t-test. This was because the data did not meet the assumptions of the t-test. When comparing frequencies to the expected probability distribution, a chi-square goodness-of-fit test was used, and Fisher’s exact test was used for comparing mortality rates. Due to the descriptive nature of this work, no hypotheses were predetermined a priori; therefore, to avoid the pitfall of type I error caused by multiple comparisons, Holm’s sequential-Bonferroni method was used to adjust for multiple testing and alpha inflation when comparing CSF findings. The null-hypothesis significance tests were two-tailed, and an alpha significance cut-off at p < 0.05 was chosen. Data were imported into and analyzed using the open-access Jamovi statistical software (version 2.2.5). The R package multxpert was used where p-value adjustments were performed for multiple comparisons.

## Results

Demographics

**Figure 2:**
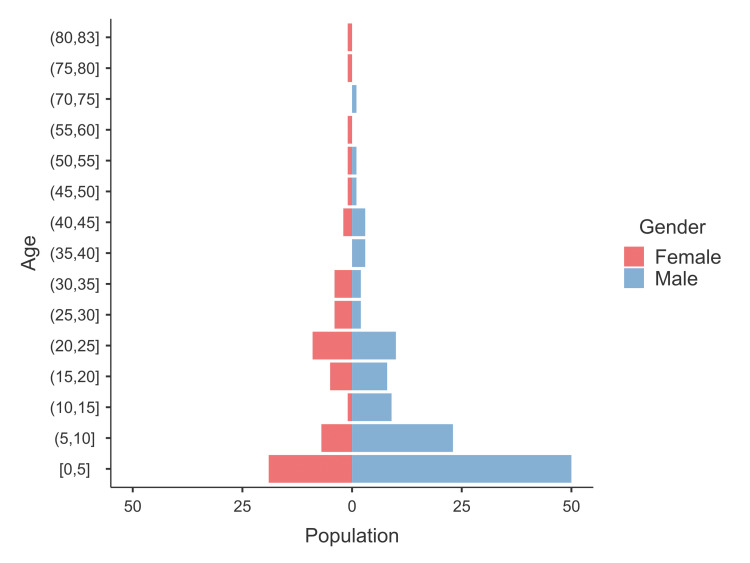
Age pyramid by years displaying the male predominance, particularly at younger ages.

A total of 169 patients met our inclusion criteria (range = one month to 83 years). Of whom, 113 (67%) were males, with a male-to-female ratio of 2, illustrating a significant male predominance (χ^2^ goodness-of-fit test, p < 0.001). Details are shown inTable 1. The occurrence was seen predominantly in the pediatric age group (118 patients, 70%), leading to a right-skewed age distribution. The median age in males was six years (1-16 years), and in females was 17.5 years (3.4-27.3 years). This difference was statistically significant (Mann-Whitney U test, p = 0.01, rank-biserial correlation effect size = 0.243, 95% confidence interval (CI) = 0.062-0.408). The frequency and age distribution differences are illustrated in Figure 2.

**Table 1:** Demographics of the studied sample. IQR: interquartile range

	Aseptic	Enteroviral	Others	Total
	(n = 83)	(n = 74)	(n = 12)	(n = 169)
Gender				
Males	55	54	4	113
Females	28	20	8	56
Age, median (IQR)				
Males	7 (0.75–23)	6 (1–8)	25 (21–28)	6 (1–16)
Females	17 (4–32)	9 (2–21)	23 (13–38)	17.5 (3.4–27.3)
Referral	27	12	4	43
Prior antibiotic treatment	40	37	7	84

Of our patients, 43 (25%) were referred from other hospitals or clinics. Aseptic meningitis did not display a particular pattern of seasonality; however, enteroviral meningitis cases peaked in May and June during the late spring season.

A total of 84 (50%) patients reported taking antibiotics prior to presentation to KAUH’s ER. The most commonly taken antibiotic was amoxicillin, with or without clavulanate (32%), followed by ceftriaxone (20%) and cefixime (10%). The mean duration of antibiotic use was 3.2 days (±2 days) prior to presentation.

Clinical features

The most commonly reported symptoms were fever and headache (Table 2). The vast majority of patients (95%) reported fever, subjective or documented. However, only 48% of patients were febrile at presentation to the ER using the threshold of 37.5°C commonly used at our center. Meningeal signs were positive in only 29 patients, and they did not have greater CSF leukocytes levels compared to those documented to have negative meningeal signs (M = 346 ± 568, N = 29 vs. M = 232 ± 352, N = 31; Mann-Whitney U test, p = 0.151, rank-biserial correlation effect size = 0.22, 95% CI = -0.07-0.47).

**Table 2:** Clinical features. *: Missing data (NA) are reported to avoid misleading percentages. ^a^:Positive of any of the three meningeal signs: Kerning, Brudzinski, and nuchal rigidity. NA: not available; ER: emergency room

Signs and symptoms	Frequency	NA*	Valid percentage (NA excluded)	Valid percent (NA included)
Fever history	148	13	95	88
Headache	106	63	100	63
Fever in the ER	81	0	48	48
Vomiting	70	98	99	41
Hypoactivity or decreased oral intake	65	104	100	38
Photophobia	41	127	98	24
Meningeal signs^a^	29	109	48	17
Nuchal rigidity	25	114	45	15
Kernig	24	114	44	14
Brudzinski	22	116	42	13
Phonophobia	21	148	1	12
Seizure or loss of consciousness	17	152	100	10
Coryza	11	158	100	7
Altered mental status	10	159	100	6
Diarrhea	8	161	100	5

CSF analysis findings

The causative agent of meningitis was identified in 86 (51%) of the 169 cases, and the remaining 83 (49%) cases were labeled as aseptic meningitis, which was the most common cause, diagnosed by exclusion. Viral PCR for CSF was positive in 80 patients. Enterovirus was the most commonly identified virus, being detected in 74 samples. HSV I or HSV II was seen in two patients, one of whom was positive for both (Table 3).

**Table 3:** Etiologies.

Etiology	Frequency	Percentage
Aseptic	83	49.1
Enterovirus	74	43.8
*Streptococcus pneumoniae*	3	1.8
Epstein-Barr virus	3	1.8
Herpes simplex virus	2	1.2
Mycobacterial	2	1.2
*Klebsiella pneumoniae*	1	0.6
Cytomegalovirus	1	0.6
Total	169	100

Cultures were positive in four patients (three *S. pneumoniae*, one *K. pneumoniae*), and *Mycobacterium tuberculosis* was identified in two patients using PCR testing, for a total of six bacterial meningitis cases (3.5%). The description of CSF findings and comparison between aseptic and enteroviral meningitis is shown in Table 4. The CSF findings of other etiologies are not included in the table, as their samples were too small to produce reliable inferences.

**Table 4:** Comparison of CSF analysis between aseptic and enteroviral meningitis. ^a^: Holm-adjusted p-values for Mann-Whitney U test. Due to the multiple testing of eight variables, alpha is inflated from 5% to 34% (familywise error rate = 1 - (1-0.05)^8^). Therefore, original p-values were multiplied sequentially by the number of comparisons (c = 8) as per the Holm procedure for multiple comparisons. This was deemed necessary to avoid type I error and alpha inflation. *: p < 0.05. CSF: cerebrospinal fluid; IQR: interquartile range

CSF parameters	Aseptic	Enteroviral	P-value^a^
	(n = 83)	(n = 74)	
Glucose (mmol/L)			
Median (IQR)	3.2 (2.9–4)	3.3 (3.0–3.7)	1
Range	0.2–7.5	1.2–5.7	
Protein (mg/dL)			
Median (IQR)	68 (40–131)	52 (31–82)	0.361
Range	1–451	1–1,148	
Erythrocytes (cells/µL)			
Median (IQR)	6.5 (1.5–81)	20.0 (5.0–113)	0.474
Range	0–16,000	0–19,200	
Leukocytes (cells/µL)			
Median (IQR)	130 (48.5–219)	191.0 (72–424)	0.049*
Range	Aug-00	10–1,870	
Lymphocytes (%)			
Median (IQR)	60 (33.0–84)	40.0 (16–66)	0.049*
Range	0–100	3–100	
Monocytes (%)			
Median (IQR)	16 (6.0–26)	16 (5–30)	1
Range	0–92	0–80	
Neutrophils (%)			
Median (IQR)	15 (2–40)	36 (10–64)	0.026*
Range	0–92	0–96	
Eosinophils (%)			
Median (IQR)	0 (0–0)	0 (0–0)	1
Range	0–21	0–11	

Regarding neutrophil predominance (i.e., ≥50%) within CSF samples, 19% of aseptic cases and 44% of enteroviral cases displayed neutrophil predominance. In CSF samples of enteroviral meningitis, neutrophil percentages were significantly higher, while lymphocyte percentages and total leukocytes were lower when compared to aseptic meningitis, as seen in Table 4. No other significant differences between the two groups were noted for CSF glucose, protein, erythrocytes, monocytes, and eosinophils.

All four bacterial cases displayed neutrophil predominance. However, in predicting bacterial etiology given that neutrophil predominance was present, the positive predictive value was 8% (4/53 = 8%). The contingency table of neutrophil predominance across bacterial and non-bacterial etiologies is shown in Table 5.

**Table 5:** CSF neutrophil predominance in bacterial and non-bacterial etiologies. ^a^:*Mycobacteria *excluded as it is expected to display lymphocyte predominance. ^b^: Both aseptic (n = 83) and enteroviral cases (n = 74). PMNs: polymorphonuclear leukocytes; CSF: cerebrospinal fluid

		Bacterial^a^	Non-bacterial^b^	Total
PMNs ≥50%	Yes	4	49	53
	No	0	108	108
	Total	4	157	161

Clinical outcomes

The outcomes for enteroviral meningitis were excellent as no deaths occurred among the 74 enteroviral cases. However, two deaths were recorded among the 83 aseptic cases, and four died out of the six bacterial cases. Mortality was significantly higher in bacterial meningitis (Fischer’s exact test, p < 0.001). No other deaths occurred.

## Discussion

Males are remarkably more represented than females in the diagnosis of meningitis. A recent meta-analysis by peer et al. [8] on national data across five countries from 1999 to 2016 identified a consistent male predominance in viral meningitis cases in every country included. This difference was driven by the higher incidence of meningitis in males under 15 years of age, while incidence rates were similar at older ages between males and females. Providing further validation in Jordan, the male-to-female ratio in our study was 3 in adolescents younger than 15 years of age, while the ratio was 1 at older ages. Prior literature from Jordan displays the same trend [9-11]. This disparity in incidence at younger ages could be attributed to the immunological sex-based differences, such as the superior immune response of females to viral infections, both through cell-mediated and humoral immunity. Additionally, estrogen stimulates further differentiation of leukocytes, while androgen suppresses leukocyte activity via anti-inflammatory cytokines [12-14].

Regarding seasonal variation, our results showed a difference from prior trends observed in our region, which have reported higher rates in the summer and peaks in July-August [10,15].

The majority of patients took antibiotics before presenting to the ER. This is due to the lack of public awareness of the perils of antibiotic misuse, in addition to the absence of monitoring of antibiotics in Jordan as they are sold over the counter in pharmacies without mandating the provision of a prescription from a licensed medical professional. In our study, oral penicillins were the most commonly used antibiotics prior to presentation, which are not used as monotherapy empirically in meningitis [4,16] and only serve to further complicate the interpretation of CSF results and reduce their diagnostic value, particularly in CSF cultures [17]. This has notable implications in affecting studies conducted in lower- and middle-income countries where antibiotic stewardship is poorly practiced because this inappropriate practice can result in missing some bacterial etiologies on culture, leading to a false diagnosis of aseptic meningitis.

The most common presenting complaints were fever, headache, vomiting, and general weakness. This is in line with prior studies which also showed that greater than 85% of patients with meningitis report fever [18]. Although the majority of patients reported a history of undocumented fever at some point during the course of their illness, only about half were febrile in the ER. This finding is of major value to clinicians because, in a patient presenting with a clinical suspicion of meningitis, the absence of fever at evaluation does not rule out the possibility of meningitis in the ER setting. Therefore, physicians should not solely use the absence of fever in the ER to exclude the necessity of lumbar puncture.

The classic physical signs of meningitis, namely, Kernig, Brudzinski, and nuchal rigidity, had very little diagnostic value. These physical signs, when combined, offered a sensitivity of only 48% when ignoring missing data and 17% when missing data were included. It is reasonable to expect the true value for sensitivity in our sample to be somewhere in between as clinicians may be more interested in positive findings. However, the specificity of these signs could not be measured as patients who did not have meningitis on lumbar puncture were not included in our study, rendering the rate of positive meningeal signs among non-meningitis patients unknown. Several studies have been conducted to assess the reliability of these signs. A systematic review of prospective studies [19] showed the meningeal signs together to have a sensitivity of 64% and a specificity of 89%. Although there is considerable variation in the values reported in different studies, the authors of those studies are consistent in their conclusion that these signs are of little to no diagnostic value and cannot be used to rule in or rule out meningitis, and that lumbar puncture is indicated if the clinical picture is suspicious for meningitis to reliably diagnose it and determine its etiologic agent [20-23]. These classic signs have been heavily emphasized in the medical curriculum for the past century and are still a hot topic in assessments and practical examinations. Multiple factors can explain the disappointing performance of these signs such as improper technique, poor interobserver agreement, and low intrinsic value of the signs per se. We should encourage a critical and evidence-based evaluation of such practices and direct attention to what future physicians will find helpful in fulfilling their responsibility of delivering safe and evidence-based diagnosis and management to their patients.

Our study also showed that the vast majority of meningitis cases outside of the neonatal period were aseptic and viral in etiology, and enterovirus was the leading viral agent, which is in line with prior regional and international reports [9,24-26]. Enteroviral cases displayed higher CSF neutrophil percentage compared to aseptic cases. This fact is of high relevance to clinicians because CSF neutrophil predominance is observed frequently in enteroviral meningitis, and, therefore, merely crossing the 50% cutoff is of weak predictive value for bacterial etiology, especially given the low base rate incidence of bacterial meningitis among other causes. Indeed, neutrophil percentages higher than half offer more specificity and greater clinical predictive value for bacterial causes. The neutrophil predominance in enteroviral meningitis is well documented [11]. A study from Korea [27] compared enteroviral meningitis with other viral etiologies and found significantly higher blood neutrophil percentages in the former. Additionally, enteroviral cases displayed lower blood and CSF lymphocyte percentages. Although they did not directly report elevated CSF neutrophils, it can be approximated as 100 minus lymphocyte percentage as the rest of the leukocyte types are of negligible proportion. The authors commented on their finding of low CSF lymphocytes in enteroviral meningitis by saying that its meaning is unclear. In-vitro and animal studies have shown that infection of neutrophils with Coxsackievirus B3, a prevalent enterovirus, triggers the release of tumor necrosis factor-alpha, interleukin-6, and other cytokines, which mediate inflammation and enhance neutrophil survival. This was implicated in the pathogenesis of viral myocarditis [28] and is a promising area of research as a better understanding of this topic in the future could lead to novel treatments for viral meningitis to alleviate the severity of symptoms or shorten the duration of illness.

The leading bacterial pathogen identified was *S. pneumoniae*. This is similar to many other trends worldwide after the introduction of the Hib vaccine [5,29]. It is unsurprising that no Hib cases were identified in our cohort as the Jordanian national vaccination program includes the Hib vaccine since its introduction in 2001. This vaccine has been shown to be highly effective and of major impact on the epidemiology of meningitis worldwide [4,5]. However, it should be noted that the widespread abuse of antibiotics by the public likely lowered the perceived culture results to some extent as prior administration of antibiotics can sterilize CSF and reduce culture growth results [17].

Putting our findings in context with prior work, local literature is unfortunately very limited. In the study by Meqdam et al. [9], enterovirus was responsible for 72% of meningitis cases and echovirus 9 was the leading serotype. Cultures were positive for bacteria in 7% of lumbar punctures, but the study did not elaborate further on bacterial etiologies. In 2004, a multinational epidemiologic study used a rapid assessment tool to study annual Hib meningitis incidence rates in children younger than five years of age [30], and it was estimated in Jordan at 14 per 100,000 children. Our study showed that Hib is no longer a common bacterial etiology, and *S. pneumoniae* is the leading bacterial etiology.

We have anecdotal evidence to suspect that Hib may reemerge to some degree due to a decrease in vaccine coverage for children during the coronavirus disease 2019 (COVID-19) pandemic lockdowns in 2020. Our clinicians have recently diagnosed the first Hib meningitis case in years, and the patient was unvaccinated. It is necessary to catch up on missed vaccinations to avoid possible future outbreaks.

We warn against the misinterpretation of the present findings. Our data on the clinical features, etiologies, and outcomes should not be generalized to populations not targeted by our study, such as neonates and patients with coexisting conditions deeming them high risk for bacterial meningitis and poorer outcomes, for example, those with craniospinal malformations, injuries, or surgeries, inserted ventriculoperitoneal shunts, and immunocompromised patients.

This study represents a milestone in the public health record of Jordan as it highlights, with good quality and clear and replicable methodology, the leading viral and bacterial etiologies of meningitis in the period preceding 2019, the year in which the pneumococcal conjugated vaccine was added to the Jordanian national vaccination program, with the aim to start administering vaccinations in 2020, although this has been unfortunately hindered by the COVID-19 pandemic, and the focusing of efforts and resources toward its vaccination. Regardless, as more children receive vaccination against *S. pneumonia* in the future, this report will be vital for future monitoring and comparison.

Study limitations

This study is limited by the frequent antibiotic abuse as some bacterial meningitis cases may have been mislabeled as aseptic, leading to the underestimation of the true number of bacterial meningitis cases. However, this also provides a more representative image of real-world patients and the clinical practice in Jordan and many countries where antibiotic misuse is prevalent. Finally, KAUH is the only tertiary care hospital in northern Jordan, and patients who were sufficiently managed in primary and secondary healthcare centers were not represented in this study.

## Conclusions

Meningitis in Jordan is most commonly aseptic and enteroviral and has excellent outcomes compared with bacterial meningitis. Enteroviral meningitis displays a higher percentage of neutrophils in CSF compared to aseptic meningitis. *S. pneumoniae* is the leading cause of bacterial meningitis. Slight neutrophil predominance above half is a weak predictor of bacterial meningitis due to the small contribution of bacteria as a cause among enteroviruses and aseptic etiologies.
